# Three-Dimensional Multi-Target Tracking Using Dual-Orthogonal Baseline Interferometric Radar

**DOI:** 10.3390/s22197549

**Published:** 2022-10-05

**Authors:** Saima Ishtiaq, Xiangrong Wang, Shahid Hassan, Alsharef Mohammad, Ahmad Aziz Alahmadi, Nasim Ullah

**Affiliations:** 1 School of Electronic and Information Engineering, Beihang University, Beijing 100191, China; 2 Department of Electrical Engineering, College of Engineering, Taif University, P.O. Box 11099, Taif 21944, Saudi Arabia

**Keywords:** MTT, 3D velocity, interferometric radar, GNN, IMM, SCKF, rule-based M/N logic

## Abstract

Multi-target tracking (MTT) generally needs either a Doppler radar network with spatially separated receivers or a single radar equipped with costly phased array antennas. However, Doppler radar networks have high computational complexity, attributed to the multiple receivers in the network. Moreover, array signal processing techniques for phased array radar also increase the computational burden on the processing unit. To resolve this issue, this paper investigates the problem of the detection and tracking of multiple targets in a three-dimensional (3D) Cartesian space based on range and 3D velocity measurements extracted from dual-orthogonal baseline interferometric radar. The contribution of this paper is twofold. First, a nonlinear 3D velocity measurement function, defining the relationship between the state of the target and 3D velocity measurements, is derived. Based on this measurement function, the design of the proposed algorithm includes the global nearest neighbor (GNN) technique for data association, an interacting multiple model estimator with a square-root cubature Kalman filter (IMM-SCKF) for state estimation, and a rule-based M/N logic for track management. Second, Monte Carlo simulation results for different multi-target scenarios are presented to demonstrate the performance of the algorithm in terms of track accuracy, computational complexity, and IMM mean model probabilities.

## 1. Introduction

**Figure 1:**
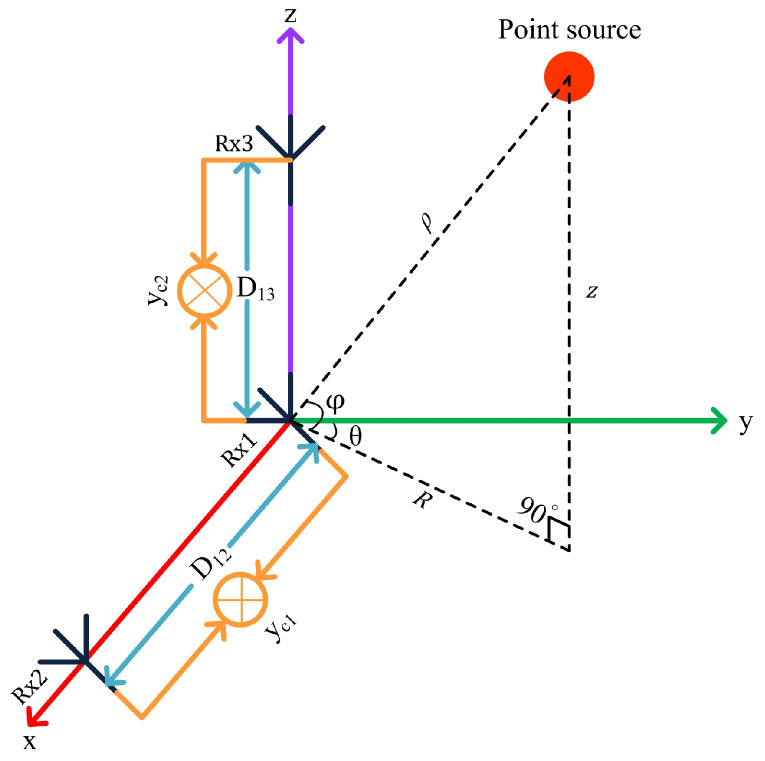
Geometry of the dual-orthogonal baseline interferometric radar with a point source in the 3D Cartesian space.

Multi-target tracking (MTT) has received increased attention in real-time systems with a broad spectrum of applications, including aircraft tracking [1], surveillance [2], remote sensing [3,4], adaptive cruise control [5], robotics [6], biomedical engineering [7], image processing [8], and oceanography [9]. The main purpose of MTT algorithms is to identify the number of potential targets in the radar’s field of view (FOV) and to estimate their kinematic states from noisy radar measurements. Various algorithms to address the problem of MTT have been proposed in the literature [10,11,12]. MTT systems generally need either a Doppler radar network with spatially separated receivers or a single radar equipped with costly phased array antennas. The Doppler radar has the capability of determining the Doppler frequency shift of the target in the radar’s FoV, which is directly related to its radial velocity [13,14,15,16]. However, Doppler radar can provide the information of range and Doppler frequency shift only. Since it is not capable of extracting the direction-of-arrival (DOA), i.e., azimuth and elevation angles, information of the target, it is not possible to localize the target in the 3D Cartesian space using the range-Doppler data from single Doppler radar receiver only [17]. To resolve this issue, a distributed Doppler radar network with multiple sensors is typically employed, which can monitor the potential target of interest from different angles spatially. Various algorithms for MTT based on Doppler radar networks exist in the literature. Multistatic radar networks have become very popular in various real-time tracking applications over time [18,19,20]. An algorithm for target tracking, by monitoring the Doppler signals at multiple spatial points employing a network of four Doppler radars, was presented in [20]. The MTT algorithm using Doppler measurements from multistatic Doppler radar with an unknown probability of detection was proposed in [21]. A technique for tracking multiple targets by exploiting the range and Doppler information from multiple radar sensors was introduced in [22]. This technique, however, demands a multitude of iterations, which may cause the system to halt and generate latency. The MTT techniques presented in [17,23] are also based on multilateration of range-Doppler data from at least three radar sensors for localizing and tracking the targets without ambiguity. Multistatic radar systems for MTT by utilizing bistatic range and range-rate information from multiple radars were described in [24,25], which send data to a central station for estimating the spatial locations and velocities of the targets in the FoV. However, the integrated hardware due to multiple radar sensors in the network requires a high data transfer rate, making it difficult to realize real-time applications. The detection and tracking system using a single radar sensor demands a phased array antenna. However, a small-scale array of antennae offers poor angular resolution. Therefore, a costly large phased array is needed to provide angular information with better resolution. Furthermore, advanced array signal processing techniques such as the ESPRIT and MUSIC algorithms essentially increase the computational burden on the data processing unit caused by the complex matrix operations [26,27]. Furthermore, the MTT algorithm using a phased array antenna requires the number of receiving elements to be greater than the number of targets in the sensor’s FoV. To resolve this issue, in our previous work [28,29], we presented an algorithm for multiple targets’ detection and tracking in the 2D Cartesian space by measuring their range and 2D velocity (radial velocity and angular velocity) measurements using a dual-frequency frequency-modulated continuous wave (DF-FMCW) interferometric radar. Now, we propose to extend this concept to MTT in the 3D Cartesian space based on 3D velocity (radial velocity, azimuth angular velocity, and elevation angular velocity) measurements using a dual-orthogonal baseline DF-FMCW interferometric radar. The geometry of the dual-orthogonal baseline interferometric radar with a point source as the target is represented in Figure 1. The reference receiving antenna Rx1 is used to extract the initial ranges and radial velocities of the targets, and two orthogonal baselines with lengths $D_{12}$ and $D_{13}$ are used to measure the azimuth angular velocities and elevation angular velocities, respectively, of the targets in the radar’s FOV. θ and φ represent the azimuth and elevation angles of the target with the y-axis and xy-plane, respectively. ρ is the range of the target relative to the observing radar in the 3D Cartesian space. The contribution of this paper is twofold:

1. First, we present a mathematical model of the 3D velocity of multiple moving point sources and derive the nonlinear 3D velocity measurement function, which defines the relationship between the state of the target in terms of the 3D Cartesian space and the 3D velocity measurements extracted from the interferometric radar. Based on this measurement function, the design and implementation of the tracking algorithm are presented, which include (i) the GNN technique combined with the auction algorithm for data association, (ii) the IMM-SCKF estimator for state estimation, and (iii) the rule-based M/N logic for track management.
2. Second, Monte Carlo simulation results with different multiple target scenarios are presented to validate the performance of the proposed algorithm in terms of track accuracy, computational complexity, and IMM mean model probabilities.

The layout of this paper is as follows. The mathematical formulation of the proposed MTT algorithm is presented in Section 2. Section 3 introduces the detailed design of the proposed MTT algorithm. The three main stages of the MTT algorithm including the GNN for data association, the IMM-SCKF estimator for state estimation, and the rule-based M/N logic for track management are described in Section 4, Section 5, Section 6 and Section 7. Section 8 presents the performance evaluation simulations. Finally, Section 9 provides the concluding remarks.

## 2. Mathematical Formulation of the Problem

The linear velocity of an arbitrary moving point source is a vector quantity. To completely localize a moving target in the 3D Cartesian space, it is necessary to measure the instantaneous 3D velocity vector $v_{i}$ of the target, which is composed of radial velocity $v_{r_{3D}}$, azimuth cross-radial velocity $v_{{cr}_{\theta }}$, and elevation cross-radial velocity, i.e., $v_{{cr}_{\varphi }}$, $v_{i}=\langle v_{r_{3D}},v_{{cr}_{\theta }},v_{{cr}_{\varphi }}\rangle$. The relationship between angular velocity and cross-radial velocity is defined as $\omega =v_{cr}/\rho$.

### 2.1. The 3D Velocity of Point Sources

Assume an FMCW radar signal with carrier frequency $f_{c}$, bandwidth *B*, and sweep time *T*. The transmitted signal can be written as

(1) $$S_{T}\left(t\right)=exp\left\{-j2\pi \left[f_{c}t_{s}+\frac{K}{2}t_{s}^{2}\right]\right\}$$

where $t_{s}=t-nT$ represents the time at the start of the *n*th sweep period and K=B⁄T is the chirp rate. For an object initially present at a range of $\rho_{0}$ and moving with radial velocity $v_{r_{3D}}$ relative to the radar in the 3D Cartesian space, the signal reflected off of the object is the same as the transmitted signal, but delayed with a round trip time $\tau_{3D}$. Therefore, the received signal at antenna Rx1 for the FMCW radar can be written as

(2) $$S_{R_{1}}\left(t\right)=exp\left\{-j2\pi \left[f_{c}\left(t_{s}-\tau_{3D}\right)+\frac{K}{2}{\left(t_{s}-\tau_{3D}\right)}^{2}\right]\right\}$$

where $\tau_{3D}=2\rho \left(t\right)/c$. The velocity of the object is considered to be slow enough that, during each pulse repetition interval, the object is generally expected to reside in the same range bin. Hence, the relative range of the target can be defined as

(3) $$\rho \left(t\right)\approx \rho \left(nt\right)=\rho_{0}+v_{r_{3D}}nT$$

According to the FMCW radar operating principle, the transmitted and received signals are mixed to obtain the beat frequency signal, which can be expressed as

(4) $$\begin{array}{cc} S_{B_{1}}\left(t\right) & =S_{T}\left(t\right)S_{R_{1}}^{*}\left(t\right)\\ \; & \approx exp\{-j4\pi \left[\frac{K\left(\rho_{0}+v_{r_{3D}}nT\right)t_{s}}{c}+\frac{\rho_{0}}{\lambda }+\frac{v_{r_{3D}}}{\lambda }nT\right]\} \end{array}$$

In the case of *M* number of point sources moving in the radar’s FOV, the beat frequency signal $S_{B_{1}}\left(t\right)$ can be expressed as

(5) $$\begin{array}{c} S_{B_{1}}\left(t\right)=\sum_{m=1}^{M}exp\left(-j4\pi \left[\frac{K\left(\rho_{0m}+v_{{rm}_{3D}}nT\right)t_{s}}{c}+\frac{\rho_{0m}}{\lambda }+\frac{v_{{rm}_{3D}}}{\lambda }nT\right]\right) \end{array}$$

where $\tau_{m_{3D}}=2\rho_{m}⁄c$ for m=1,2,⋯,M. The relative radial motion of the object induces a Doppler frequency shift in the radar’s received signal. The radial velocity in terms of the Doppler frequency shift can be defined as

(6) $$v_{{rm}_{3D}}=\left[\frac{f_{{dm}_{3D}}\lambda }{2}=\frac{cf_{{dm}_{3D}}}{2f_{c}},\;m=1,2,\cdots ,M\right]$$

The first baseline, constituting the receiving antennas Rx1 and Rx2 along the x-axis, is used to measure the azimuth angular velocity of the targets in the radar’s FOV. Considering the signal received by antenna Rx1 as the reference, the signal received by antenna Rx2, placed at a geometrical distance of $D_{12}$, can be represented as

(7) $$\begin{array}{c} S_{R_{2}}\left(t\right)=exp\left\{-j2\pi \left[f_{c}\left(t_{s}-\tau_{3D}-\tau_{0_{\theta }}\right)+\frac{K}{2}{\left(t_{s}-\tau_{3D}-\tau_{0_{\theta }}\right)}^{2}\right]\right\} \end{array}$$

where $\tau_{0_{\theta }}=D_{12}\sin\theta /c$. The second beat frequency signal $S_{B_{2}}\left(t\right)$ at receiving antenna Rx2 can be written as

(8) $$\begin{array}{cc} S_{B_{2}}\left(t\right) & =S_{T}\left(t\right)S_{R_{2}}^{*}\left(t\right)\\ \; & \approx exp\{-j2\pi \left[\frac{2K\left(\rho_{0}+v_{r_{3D}}nT\right)t_{s}}{c}+\frac{2v_{r_{3D}}nT}{\lambda }+\frac{2\rho_{0}}{\lambda }+\left(f_{c}+Kt_{s}\right)\frac{D_{12}\sin\theta }{c}\right]\} \end{array}$$

Following the interferometric radar principle, the beat frequency signals $S_{B_{1}}\left(t\right)$ and $S_{B_{2}}$ at the two receiving antennas are correlated to generate the interferometric output [30]:

(9) $$\begin{array}{cc} y_{c_{1}}\left(t\right) & =S_{B_{1}}\left(t\right)S_{B_{2}}^{*}\left(t\right)\\ \; & =exp\{j2\pi \left(f_{c}+Kt_{s}\right)\frac{D_{12}\sin\theta }{c}\} \end{array}$$

In the case of *M* number of moving point sources, the interferometric output $y_{c_{1}}\left(t\right)$ represented by Equation (9) can be re-written as

(10) $$\begin{array}{cc} y_{c_{1}}\left(t\right) & =\sum_{m,b=1}^{M}exp\left(-j4\pi \left[\frac{K\left(\rho_{0m}+v_{{rm}_{3D}}nT\right)t_{s}}{c}+\frac{\rho_{0m}}{\lambda }+\frac{v_{{rm}_{3D}}nT}{\lambda }\right]\right)\\ & exp(j2\pi \left[\frac{2K\left(\rho_{0b}+v_{{rb}_{3D}}nT\right)t_{s}}{c}+\frac{2v_{{rb}_{3D}}nT}{\lambda }+\frac{2\rho_{0b}}{\lambda }+\left(f_{c}+Kt_{s}\right)\frac{D_{12}\sin\theta_{b}}{c}\right])\\ & =\sum_{m=1}^{M}exp\left(j2\pi \left[f_{c}+Kt_{s}\right]\frac{D_{12}\sin\theta_{m}}{c}\right)\\ & \sum_{m=1}^{M}\sum_{b=1,m\neq b}^{M}exp(-j4\pi [\frac{\left(\rho_{0m}+v_{rm_{3D}}nT\right)-\left(\rho_{0b}+v_{{rb}_{3D}}nT\right)}{c}Kt_{s}\\ & +\frac{\left(v_{{rm}_{3D}}nT-v_{{rb}_{3D}}nT\right)}{\lambda }+\frac{\left(\rho_{0m}-\rho_{0b}\right)}{\lambda }-\left(f_{c}+Kt_{s}\right)\frac{D_{12}\sin\theta_{b}}{2c}]) \end{array}$$

As can be seen from Equation (10), the interferometric output is composed of two parts. The first part consists of *M* intra-correlation terms, generated by the angular velocities of moving objects, whereas the second part consists of M(M−1) nuisance inter-correlation terms in which the radial and angular velocities of different moving objects are coupled together. In order to extract the angular velocities of the objects, these intermodulation terms need to be suppressed. Once these intermodulation terms are suppressed, azimuth angular velocity $\omega_{\theta }$ in terms of azimuth interferometric frequency shift $f_{\theta }$ is defined as

(11) $$\omega_{\theta m}=\frac{tial\theta_{m}}{tialt}=\left[\frac{f_{\theta m}\lambda_{t_{s}}}{D_{12}},\;m=1,2,\cdots ,M\right]$$

The second orthogonal baseline, constituting the receiving antennas Rx1 and Rx3 along the z-axis separated by geometrical distance $D_{13}$, is used to measure the elevation angular velocity of the targets in the radar’s FOV. Now, considering the signal received by the first receiving antenna Rx1 as the reference, the signal received by receiving antenna Rx3 can be written as

(12) $$\begin{array}{cc} S_{R_{3}}\left(t\right) & =exp\{-j2\pi \left[f_{c}\left(t_{s}-\tau_{3D}-\tau_{0_{\varphi }}\right)+\frac{K}{2}{\left(t_{s}-\tau_{3D}-\tau_{0_{\varphi }}\right)}^{2}\right]\} \end{array}$$

where time delay $\tau_{0_{\varphi }}=D_{13}\sin\varphi /c$. The transmitted and received signals represented by Equations (1) and (12), respectively, are mixed to generate the beat frequency signal. That is,

(13) $$\begin{array}{cc} S_{B_{3}}\left(t\right) & =S_{T}\left(t\right)S_{R_{3}}^{*}\left(t\right)\\ \; & \approx exp\{-j2\pi \left[\frac{2K\left(\rho_{0}+v_{r_{3D}}nT\right)t_{s}}{c}+\frac{2v_{r_{3D}}nT}{\lambda }+\frac{2\rho_{0}}{\lambda }+\left(f_{c}+Kt_{s}\right)\frac{D_{13}\sin\varphi }{c}\right]\} \end{array}$$

The interferometric output, $y_{c_{2}}\left(t\right)$, of the second baseline along the z-axis is

(14) $$\begin{array}{cc} y_{c_{2}}\left(t\right) & =S_{B_{1}}\left(t\right)S_{B_{3}}^{*}\left(t\right)\\ \; & =exp\{j2\pi \left(f_{c}+Kt_{s}\right)\frac{D_{13}\sin\varphi }{c}\} \end{array}$$

In the case of *M* number of point sources moving in the radar’s FOV, the interferometric output $y_{c_{2}}\left(t\right)$ takes the following form.

(15) $$\begin{array}{cc} y_{c_{2}}\left(t\right) & =\sum_{m,b=1}^{M}exp\left(-j4\pi \left[\frac{K\left(\rho_{0m}+v_{{rm}_{3D}}nT\right)t_{s}}{c}+\frac{\rho_{0m}}{\lambda }+\frac{v_{{rm}_{3D}}nT}{\lambda }\right]\right)\\ & exp(j2\pi \left[\frac{2K\left(\rho_{0b}+v_{{rb}_{3D}}nT\right)t_{s}}{c}+\frac{2v_{{rb}_{3D}}nT}{\lambda }+\frac{2\rho_{0b}}{\lambda }+(f_{c}+Kt_{s}\right)\frac{D_{13}\sin\varphi_{b}}{c}])\\ & =\sum_{m=1}^{M}exp\left(j2\pi \left[f_{c}+Kt_{s}\right]\frac{D_{13}\sin\varphi_{m}}{c}\right)\\ & \sum_{m=1}^{M}\sum_{b=1,m\neq b}^{M}exp(-j4\pi [\frac{\left(\rho_{0m}+v_{{rm}_{3D}}nT\right)-\left(\rho_{0b}+v_{{rb}_{3D}}nT\right)}{c}Kt_{s}\\ & +\frac{\left(v_{{rm}_{3D}}nT-v_{{rb}_{3D}}nT\right)}{\lambda }+\frac{\left(\rho_{0m}-\rho_{0b}\right)}{\lambda }-\left(f_{c}+Kt_{s}\right)\frac{D_{13}\sin\varphi_{b}}{2c}]) \end{array}$$

Similar to the case of azimuth angular velocity measurement, the interferometric output $y_{c_{2}}\left(t\right)$ also consists of two parts, including *M* intra-correlation terms and M(M−1) inter-correlation terms. The inter-correlation terms interfere with the extraction of the elevation angular velocities of the objects. The elevation angular velocity $\omega_{\varphi }$, in terms of elevation interferometric frequency shift $f_{\varphi }$, is defined as

(16) $$\omega_{\varphi m}=\frac{tial\varphi_{m}}{tialt}=\left[\frac{f_{\varphi m}\lambda_{t_{s}}}{D_{13}},\;m=1,2,\cdots ,M\right]$$

### 2.2. Process Model

The process model describes the state transition between two consecutive time instants. Consider modeling the motion of the target by one of the *i* hypothesis models. These models can be represented by a set as $M_{r}:=\left\{1,2,\cdots ,r\right\}$. $M_{k-1}^{j}$ represents the event of model *j* being effective during time period $\left(t_{k-1},t_{k}\right]$. In this case, the target dynamic/motion for the *j*th hypothesis model can be described as

(17) $$x_{k}=F_{k-1}^{j}x_{k-1}+v_{k-1}^{j}$$

where $F_{k-1}^{j}$ represents the state transition matrix for motion model *j* being effective at time k−1, $x_{k}$ represents the target’s state vector at time *k*, and $v_{k-1}^{j}$ represents the process noise for the *j*th dynamic model, which is assumed to be independent and identically distributed (i.i.d.) zero-mean Gaussian noise with covariance $Q_{k-1}^{j}$, such that $v_{k-1}^{j}∼N\left(0,Q_{k-1}^{j}\right)$. The target motion in the 3D Cartesian space is modeled with the nearly constant velocity (NCV) and nearly coordinated turn (NCT) process models. For uniform motion, the discrete-time NCV state dynamics combined with the DWNA model are represented by

(18) $$x_{k}=F_{k-1}^{\mathrm{NCV}}x_{k-1}+\Gamma_{1}v_{1_{k-1}}$$

The state vector of the target with $n_{x}=6$ is defined as

(19) $$x_{k}={\left[x_{k},v_{x_{k}},y_{k},v_{y_{k}},z_{k},v_{z_{k}}\right]}^{⊤}$$

where $x_{k},y_{k},z_{k}$ and $v_{x_{k}},v_{y_{k}},v_{z_{k}}$ denote the Cartesian coordinates and velocities of the object, respectively, at time instant *k*. $n_{x}$ represents the state vector dimension. The state transition matrix $F^{\mathrm{NCV}}$ for the NCV model is

(20) $$\begin{array}{c} F_{k-1}^{\mathrm{NCV}}=\left[\begin{array}{cccccc} 1 & \Delta T & 0 & 0 & 0 & 0\\ 0 & 1 & 0 & 0 & 0 & 0\\ 0 & 0 & 1 & \Delta T & 0 & 0\\ 0 & 0 & 0 & 1 & 0 & 0\\ 0 & 0 & 0 & 0 & 1 & \Delta T\\ 0 & 0 & 0 & 0 & 0 & 1 \end{array}\right] \end{array}$$

The noise gain $\Gamma_{1}$ can be written as

(21) $$\begin{array}{c} \Gamma_{1}=\left[\begin{array}{ccc} \frac{\Delta T^{2}}{2} & 0 & 0\\ \Delta T & 0 & 0\\ 0 & \frac{\Delta T^{2}}{2} & 0\\ 0 & \Delta T & 0\\ 0 & 0 & \frac{\Delta T^{2}}{2}\\ 0 & 0 & \Delta T \end{array}\right] \end{array}$$

The covariance of the process noise multiplied by gain $\Gamma_{1}$ is

(22) $$\begin{array}{c} Q_{1_{k-1}}=\left[\begin{array}{cccccc} \frac{\Delta T^{4}}{4} & \frac{\Delta T^{3}}{2} & 0 & 0 & 0 & 0\\ \frac{\Delta T^{3}}{2} & \Delta T^{2} & 0 & 0 & 0 & 0\\ 0 & 0 & \frac{\Delta T^{4}}{4} & \frac{\Delta T^{3}}{2} & 0 & 0\\ 0 & 0 & \frac{\Delta T^{3}}{2} & \Delta T^{2} & 0 & 0\\ 0 & 0 & 0 & 0 & \frac{\Delta T^{4}}{4} & \frac{\Delta T^{3}}{2}\\ 0 & 0 & 0 & 0 & \frac{\Delta T^{3}}{2} & \Delta T^{2} \end{array}\right]\sigma_{v_{1}}^{2} \end{array}$$

where $\sigma_{v_{1}}^{2}$ represents the variance of process noise $v_{1}$. Here, $v_{1}$ is a zero-mean Gaussian white noise used to model small accelerations, with an appropriate covariance $Q_{1_{k-1}}$, which is a design parameter. For modeling the target maneuver, the discrete-time NCT state dynamics combined with the DWNA model in the 3D Cartesian coordinate system are represented by

(23) $$x_{k}=F_{k-1}^{\mathrm{NCT}}x_{k-1}+\Gamma_{2}v_{2_{k-1}}$$

The state vector of the target augmented by turn rate Ω, i.e., $n_{x}=7$, is defined as

(24) $$x_{k}={\left[x_{k},v_{x_{k}},y_{k},v_{y_{k}},z_{k},v_{z_{k}},\Omega_{k}\right]}^{⊤}$$

The state transition matrix $F^{\mathrm{NCT}}$ for the NCT model is

(25) $$\begin{array}{c} F_{k-1}^{\mathrm{NCT}}=\left[\begin{array}{ccccccc} 1 & \frac{\sin(\Omega_{k-1}\Delta T)}{\Omega_{k-1}} & 0 & \frac{\cos(\Omega_{k-1}\Delta T)-1}{\Omega_{k-1}} & 0 & 0 & 0\\ 0 & \cos(\Omega_{k-1}\Delta T) & 0 & -\sin(\Omega_{k-1}\Delta T) & 0 & 0 & 0\\ 0 & \frac{1-\cos(\Omega_{k}\Delta T)}{\Omega_{k-1}} & 1 & \frac{\sin(\Omega_{k-1}\Delta T)}{\Omega_{k-1}} & 0 & 0 & 0\\ 0 & \sin(\Omega_{k-1}\Delta T) & 0 & \cos(\Omega_{k-1}\Delta T) & 0 & 0 & 0\\ 0 & 0 & 1 & 0 & 0 & \Delta T & 0\\ 0 & 0 & 0 & 0 & 0 & 1 & 0\\ 0 & 0 & 0 & 0 & 0 & 0 & 1 \end{array}\right] \end{array}$$

The noise gain $\Gamma_{2}$ for the DWNA model is defined as

(26) $$\begin{array}{c} \Gamma_{1}=\left[\begin{array}{cccc} \frac{\Delta T^{2}}{2} & 0 & 0 & 0\\ \Delta T & 0 & 0 & 0\\ 0 & \frac{\Delta T^{2}}{2} & 0 & 0\\ 0 & \Delta T & 0 & 0\\ 0 & 0 & \frac{\Delta T^{2}}{2} & 0\\ 0 & 0 & \Delta T & 0\\ 0 & 0 & 0 & \Delta T \end{array}\right] \end{array}$$

The covariance of the process noise multiplied by gain $\Gamma_{2}$ is

(27) $$\begin{array}{c} Q_{2_{k-1}}=\left[\begin{array}{ccccccc} \frac{\Delta T^{4}}{4} & \frac{\Delta T^{3}}{2} & 0 & 0 & 0 & 0 & 0\\ \frac{\Delta T^{3}}{2} & \Delta T^{2} & 0 & 0 & 0 & 0 & 0\\ 0 & 0 & \frac{\Delta T^{4}}{4} & \frac{T^{3}}{2} & 0 & 0 & 0\\ 0 & 0 & \frac{\Delta T^{3}}{2} & \Delta T^{2} & 0 & 0 & 0\\ 0 & 0 & 0 & 0 & \frac{\Delta T^{4}}{4} & \frac{\Delta T^{3}}{2} & 0\\ 0 & 0 & 0 & 0 & \frac{\Delta T^{3}}{2} & \Delta T^{2} & 0\\ 0 & 0 & 0 & 0 & 0 & 0 & \Delta T^{2} \end{array}\right]\sigma_{v_{2}}^{2} \end{array}$$

where $\sigma_{v_{2}}^{2}$ represents the variance of process noise $v_{2}$.

### 2.3. Nonlinear Measurement Model

The nonlinear measurement model defining the relationship between the 3D velocity measurements received from the interferometric radar and the state of the target is defined as

(28) $$z_{k}=h\left(x_{k}\right)+w_{k}$$

where $h(x_{k})$ is the nonlinear 3D velocity measurement function and $z_{k}={\left[v_{r_{3D,k}},\omega_{\theta_{k}},\omega_{\varphi_{k}}\right]}^{⊤}$ with $n_{z}=3$. Here, $n_{z}$ denotes the dimension of the measurement vector. $w_{k}$ is assumed to be an i.i.d. zero-mean Gaussian measurement noise with covariance $R_{k}$, i.e., $w_{k}∼N\left(0,R_{k}\right)$ and $E\{v_{k}w_{k}^{T}\}=0$.

#### Derivation of 3D Velocity Measurement Function

The range of the target relative to the observing radar in the 3D Cartesian space is expressed as

(29) $$\rho_{k}=\sqrt{{\left(x_{k}-x_{s}\right)}^{2}+{\left(y_{k}-y_{s}\right)}^{2}+{\left(z_{k}-z_{s}\right)}^{2}}$$

By taking the time derivative of Equation (29), the radial velocity of the target in the 3D Cartesian space can be determined. That is,

(30) $${\dot{\rho }}_{k}=\frac{d\rho }{dt}=\frac{\left(x_{k}-x_{s}\right){\dot{x}}_{k}+\left(y_{k}-y_{s}\right){\dot{y}}_{k}+\left(z_{k}-z_{s}\right){\dot{z}}_{k}}{\sqrt{{\left(x_{k}-x_{s}\right)}^{2}+{\left(y_{k}-y_{s}\right)}^{2}+{\left(z_{k}-z_{s}\right)}^{2}}}$$

As we know that $v_{r_{3D,k}}={\dot{\rho }}_{k}$, $v_{x_{k}}={\dot{x}}_{k}$, $v_{y_{k}}={\dot{y}}_{k}$, and $v_{z_{k}}={\dot{z}}_{k}$, the expression for the radial velocity can be written as

(31) $$v_{r_{3D,k}}={\dot{\rho }}_{k}=\frac{\left(x_{k}-x_{s}\right)v_{x_{k}}+\left(y_{k}-y_{s}\right)v_{y_{k}}+\left(z_{k}-z_{s}\right)v_{z_{k}}}{\sqrt{{\left(x_{k}-x_{s}\right)}^{2}+{\left(y_{k}-y_{s}\right)}^{2}+{\left(z_{k}-z_{s}\right)}^{2}}}$$

According to [29], the azimuth angular velocity is determined by

(32) $$\omega_{\theta_{k}}={\dot{\theta }}_{k}=\left[\frac{\left(y_{k}-y_{s}\right)v_{x_{k}}-\left(x_{k}-x_{s}\right)v_{y_{k}}}{{\left(x_{k}-x_{s}\right)}^{2}+{\left(y_{k}-y_{s}\right)}^{2}}\right]$$

The elevation angle $\varphi_{k}$ relative to the observing radar is defined as

(33) $$\varphi =\tan^{-1}\frac{\left(z_{k}-z_{s}\right)}{R_{k}}$$

Now, by re-arranging and taking the time derivative of Equation (33),

(34) $$\left(\sec^{2}\varphi_{k}\right)\dot{\varphi }=\frac{R_{k}{\dot{z}}_{k}-\left(z_{k}-z_{s}\right){\dot{R}}_{k}}{{R_{k}}^{2}}$$

(35) $$\omega_{\varphi_{k}}=\left[\frac{R_{k}^{2}v_{z_{k}}-\left(z_{k}-z_{s}\right)\left\{\left(x_{k}-x_{s}\right)v_{x_{k}}+\left(y_{k}-y_{s}\right)v_{y_{k}}\right\}}{R_{k}^{3}}\right]\left(\cos^{2}\varphi_{k}\right)$$

where $\omega_{\varphi_{k}}={\dot{\varphi }}_{k}$. Since we know that

(36) $$\cos\varphi_{k}=\frac{R_{k}}{\rho_{k}}$$

Equation (35) can be re-written as

(37) $$\omega_{\varphi_{k}}={\dot{\varphi }}_{k}=\left[\frac{R_{k}^{2}v_{z_{k}}-\left(z_{k}-z_{s}\right)\left\{\left(x_{k}-x_{s}\right)v_{x_{k}}+\left(y_{k}-y_{s}\right)v_{y_{k}}\right\}}{R_{k}^{3}}\right]\left[\frac{R^{2}}{\rho_{k}^{2}}\right]$$

(38) $$\omega_{\varphi_{k}}=\left[\frac{R_{k}^{2}v_{z_{k}}-\left(x_{k}-x_{s}\right)\left(z_{k}-z_{s}\right)v_{x_{k}}-\left(y_{k}-y_{s}\right)\left(z_{k}-z_{s}\right)v_{y_{k}}}{\rho_{k}^{2}R_{k}}\right]$$

Proceeding from Equations (31), (32) and (38), the nonlinear 3D velocity measurement function can be defined as

(39) $$\begin{array}{c} h\left(x_{k}\right)=\left[\begin{array}{c} \frac{\left(x_{k}-x_{s}\right)v_{x_{k}}+\left(y_{k}-y_{s}\right)v_{y_{k}}+\left(z_{k}-z_{s}\right)v_{z_{k}}}{\sqrt{{\left(x_{k}-x_{s}\right)}^{2}+{\left(y_{k}-y_{s}\right)}^{2}+{\left(z_{k}-z_{s}\right)}^{2}}}\\ \frac{\left(y_{k}-y_{s}\right)v_{x_{k}}-\left(x_{k}-x_{s}\right)v_{y_{k}}}{{\left(x_{k}-x_{s}\right)}^{2}+{\left(y_{k}-y_{s}\right)}^{2}}\\ \frac{R_{k}^{2}v_{z_{k}}-\left(x_{k}-x_{s}\right)\left(z_{k}-z_{s}\right)v_{x_{k}}-\left(y_{k}-y_{s}\right)\left(z_{k}-z_{s}\right)v_{y_{k}}}{\rho_{k}^{2}R_{k}} \end{array}\right] \end{array}$$

Hence, the nonlinear measurement model defined by Equation (28) takes on the following form.

(40) $$\begin{array}{c} z_{k}=\left[\begin{array}{c} \frac{\left(x_{k}-x_{s}\right)v_{x_{k}}+\left(y_{k}-y_{s}\right)v_{y_{k}}+\left(z_{k}-z_{s}\right)v_{z_{k}}}{\sqrt{{\left(x_{k}-x_{s}\right)}^{2}+{\left(y_{k}-y_{s}\right)}^{2}+{\left(z_{k}-z_{s}\right)}^{2}}}\\ \frac{\left(y_{k}-y_{s}\right)v_{x_{k}}-\left(x_{k}-x_{s}\right)v_{y_{k}}}{{\left(x_{k}-x_{s}\right)}^{2}+{\left(y_{k}-y_{s}\right)}^{2}}\\ \frac{R_{k}^{2}v_{z_{k}}-\left(x_{k}-x_{s}\right)\left(z_{k}-z_{s}\right)v_{x_{k}}-\left(y_{k}-y_{s}\right)\left(z_{k}-z_{s}\right)v_{y_{k}}}{\rho_{k}^{2}R_{k}} \end{array}\right]+w_{k} \end{array}$$

The objective of the proposed MTT algorithm is to estimate the state ${\hat{x}}_{k|k}=E\left\{x_{k}|z_{k}\right\}$ and error covariance $P_{k|k}=E\left\{\left[x_{k}-{\hat{x}}_{k|k}\right]{\left[x_{k}-{\hat{x}}_{k|k}\right]}^{⊤}|z_{k}\right\}$ of each target in the radar’s FOV by exploiting the derived 3D velocity measurement function.

## 3. Design of the Proposed 3D MTT Algorithm

**Figure 2:**
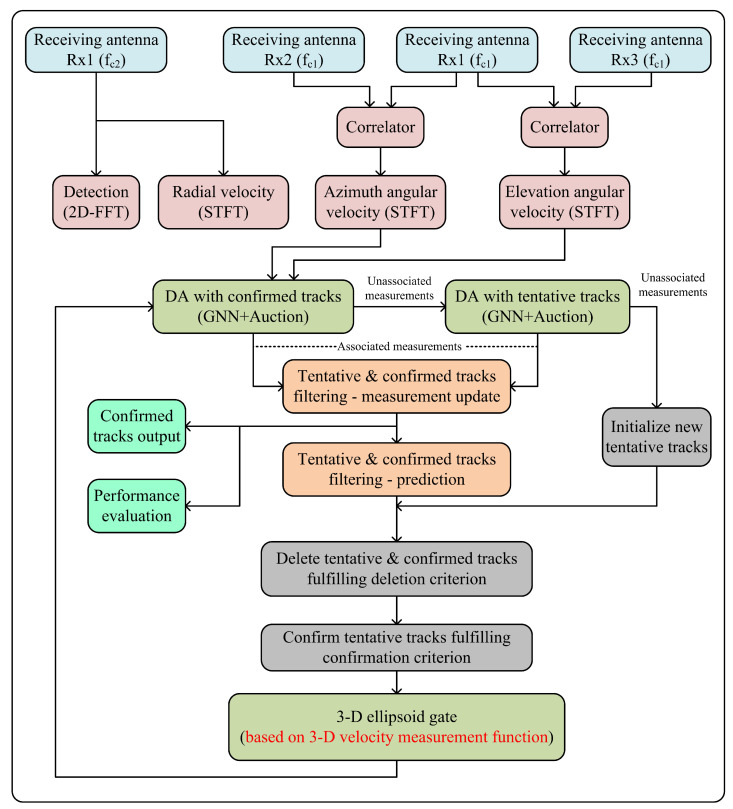
Illustration of the proposed MTT algorithm based on 3D velocity measurements obtained from the dual-frequency dual-orthogonal baseline interferometric radar.

The detailed design of the proposed MTT algorithm based on 3D velocity measurements is delineated in Figure 2. The dual-orthogonal baseline DF-FMCW interferometric radar used to measure the 3D velocities of multiple moving objects has the capability to transmit FMCW signals with two different carrier frequencies, such that $f_{c_{1}}=6$ GHz and $f_{c_{2}}=24$ GHz. It consists of one transmitting antenna Tx and three receiving antennas Rx1, Rx2, and Rx3. The transmitting antenna Tx and receiving antenna Rx1 can switch between two operating frequencies $f_{c_{1}}$ and $f_{c_{2}}$ via RF switches, depending on the mode of operation. Based on the carrier frequency $f_{c_{1}}$, the lengths of the two orthogonal baselines were set as $D_{12}=D_{13}=60\lambda_{c_{1}}=3$ m. The detection and extraction of radial velocity measurements were performed at a higher carrier frequency $f_{c_{2}}$, whereas the azimuth and elevation angular velocity measurements were obtained at a lower carrier frequency $f_{c_{1}}$, thereby suppressing the intermodulation terms in the interferometric response. The interferometric frequencies’ information was preserved by increasing the baseline lengths $D_{12}$ and $D_{13}$ simultaneously [28]. First of all, the radar observes the region of interest (ROI) operating at carrier frequency $f_{c_{2}}$ and performs 2D-FFT on the received data for the detection of the potential targets by obtaining the range–radial velocity map. Once the targets are identified, STFT is applied to the received data, which provides the time-varying Doppler spectrogram of the targets in the FOV. Following Equation (6), the radial velocities of the targets are extracted from the Doppler spectrogram [29]. Then, for the azimuth and elevation angular velocity measurements at carrier frequency $f_{c_{1}}$, STFT is performed on the two interferometric outputs $y_{c_{1}}\left(t\right)$ and $y_{c_{2}}\left(t\right)$, respectively. The time-varying azimuth and elevation interferometric spectrograms thus obtained are used to calculate the azimuth and elevation angular velocities of the targets following Equations (11) and (16), respectively. In order to combine the 3D velocity measurements of each object, 2D FFT is performed on the three beat frequency signals at antennas Rx1, Rx2, and Rx3 in the interferometric mode corresponding to the carrier frequency $f_{c_{1}}$. The following relationships hold true in the interferometric mode.

(41) $$\rho_{2}-\rho_{1}=D_{12}\sin\theta$$

(42) $$\rho_{3}-\rho_{1}=D_{13}\sin\varphi$$

where $\rho_{1}$, $\rho_{2}$, and $\rho_{3}$ represent the ranges of the object relative to antennas Rx1, Rx2, and Rx3, respectively. By taking the time derivative of Equations (41) and (42), the relationship between the radial and angular velocities of the object can be written as

(43) $$v_{r_{3D,2}}-v_{r_{3D,1}}=D_{12}\omega_{\theta }\cos\theta$$

(44) $$v_{r_{3D,3}}-v_{r_{3D,1}}=D_{13}\omega_{\varphi }\cos\varphi$$

where $v_{r_{3D,1}}$, $v_{r_{3D,2}}$, and $v_{r_{3D,2}}$ represent the radial velocity of the object at antennas Rx1, Rx2, and Rx3, respectively. The initial range measurements obtained by performing 2D FFT on the beat frequency signals are used to calculate the initial azimuth and elevation angles of the objects following Equations (41) and (42). Then, based on Equations (43) and (44), the initial values of the radial velocities along with the azimuth and elevation angle measurements are used to combine the 3D velocities of each target distinctly. Measurement-to-track data association and target state estimation are the two fundamental elements of the MTT algorithm. Moreover, the track management is also a crucial element of the MTT algorithm, which maintains the two major data structures, namely tentative and confirmed track lists. The track management unit handles the initiation of new target tracks, the confirmation of tentative tracks, and the deletion of tentative and confirmed tracks based on some predefined criteria. After the formation of the tentative and confirmed track lists from previous scans of the data, the new 3D velocity measurements received from the interferometric radar are assessed for association with existing target tracks or for initializing new tentative tracks. Based on the 3D velocity measurement function defined by Equation (39), the global nearest neighbor (GNN) method combined with the auction algorithm is used for measurement-to-track data association. The unassociated measurements are then tested for association with already existing tentative tracks. The measurements still unassociated with any existing tracks are then used to initiate new tracks. The 3D velocity measurements associated with the existing tracks are utilized in the filter measurement update step for the target’s state estimation. Since Equation (39) clearly demonstrates the nonlinear relation between the state of the target and the radar measurements, the proposed MTT algorithm utilizes the nonlinear IMM-SCKF estimator. Based on the 3D velocity measurement function, IMM-SCKF estimator is used to estimate the states of non-maneuvering and maneuvering targets. Different blocks of the MTT algorithm are thoroughly discussed in the following sections for better understanding of the algorithm.

## 4. Global Nearest Neighbor Data Association Method

Various data association techniques exist in the literature for the MTT algorithms, which include the NN, GNN, JPDA, and MHT algorithms. However, the GNN is the most commonly used optimal and robust technique for practical tracking systems. The GNN algorithm propagates a single global hypothesis and addresses the measurement-to-track association in the form of an optimal assignment problem [29,31,32]. Based on the 3D velocity measurement function expressed by Equation (39), the data association process follows three steps:

1. Ellipsoid gating;
2. GNN-algorithm-based assignment matrix formation;
3. Auction algorithm for the optimal solution of the assignment matrix.

Gating is a technique for eliminating unlikely observation-to-track pairings. For a measurement to satisfy the ellipsoid gating relationship of a track, the norm of the measurement residual vector $d_{k}^{2}$ must fulfill the following criterion:

(45) $$d_{k}^{2}={\tilde{v}}_{k}^{⊤}S_{Z,k}^{-1}{\tilde{v}}_{k}\leq G$$

where $\tilde{v}$ is the measurement residual vector, $S_{Z,k}$ is the measurement residual covariance matrix at time instant *k*, and *G* represents the gate size. Based on the 3D velocity measurement function, the measurement residual vector can be expressed as

(46) $${\tilde{v}}_{k}=z_{k}-\left[\begin{array}{c} \frac{\left(x_{k}-x_{s}\right)v_{x_{k}}+\left(y_{k}-y_{s}\right)v_{y_{k}}+\left(z_{k}-z_{s}\right)v_{z_{k}}}{\sqrt{{\left(x_{k}-x_{s}\right)}^{2}+{\left(y_{k}-y_{s}\right)}^{2}+{\left(z_{k}-z_{s}\right)}^{2}}}\\ \frac{\left(y_{k}-y_{s}\right)v_{x_{k}}-\left(x_{k}-x_{s}\right)v_{y_{k}}}{{\left(x_{k}-x_{s}\right)}^{2}+{\left(y_{k}-y_{s}\right)}^{2}}\\ \frac{R_{k}^{2}v_{z_{k}}-\left(x_{k}-x_{s}\right)\left(z_{k}-z_{s}\right)v_{x_{k}}-\left(y_{k}-y_{s}\right)\left(z_{k}-z_{s}\right)v_{y_{k}}}{\rho_{k}^{2}R_{k}} \end{array}\right]{\hat{x}}_{k|k-1}$$

Generally, $d^{2}$ is assumed to have a chi distribution ($\chi_{n_{z}}^{2}$) for correct measurement-to-track association, where $n_{z}$ represents the dimension of the measurement vector. The relationship between the gate size *G* and the probability of the 3D measurement vector falling inside the gate $P_{G}\left(n_{z}\right)$ is expressed as [11]

(47) $$P_{G}\left(3\right)=2gc\left(\sqrt{G^{3D}}\right)-\sqrt{\frac{2G^{3D}}{\pi }}exp\left(\frac{-G^{3D}}{2}\right)$$

where gc denotes standard Gaussian probability integral. Following the ellipsoid gating, the GNN algorithm is used to resolve measurement-to-track association conflicts by forming and solving a 2D assignment matrix [11]. For the formation of the assignment matrix, the rows are occupied by 3D velocity measurements, and the first columns of the assignment matrix are filled with the existing tracks. The rest of the columns are filled with new track hypotheses. The entries of the assignment matrix are expressed in the form of the score gain. That is,

(48) $$\alpha_{ij}=G-d_{ij}^{2}$$

where $\alpha_{ij}$ represents the margin by which the statistical distance passed the gate. The entries corresponding to the assignment of a measurement to a new track were set as zero. The non-allowed measurements are represented by −∞. The optimal solution is the set of assignments that produces the maximum score gain [11]. In the literature, various algorithms exist to solve the assignment problem, such as the Hungarian algorithm, the Munkres algorithm [33], the Jonker–Volgenant–Castanon (JVC) algorithm, and the auction algorithm [11]. However, the auction algorithm is presently the most efficient algorithm for the assignment problem, which has replaced the Munkres algorithm [12]. Therefore, in the current work, the GNN method with the auction algorithm was used for measurement-to-track data association.

## 5. IMM-SCKF Estimator for State Estimation

The main function of the estimation filter in the MTT algorithm is to estimate the state of the targets detected from noise-corrupted radar measurements [34]. The measurements allocated to the tracks present in the tentative or confirmed track lists during the data association step are utilized in the measurement update stage of the filtering procedure [24]. Referring to Equation (39), it is clear that there exists nonlinear relation between the 3D velocity measurements received from the interferometric radar and the state of the target. Therefore, a nonlinear filter is required for estimating the state of the target [31,32,35].

### 5.1. Square-Root Cubature Kalman Filter

Based on the properties of symmetry and positive semi-definiteness, the nonlinear SCKF is used for target state estimation. It is numerically more accurate, stable, and computationally efficient compared to the extended Kalman filter (EKF) [36], unscented Kalman filter (UKF) [37], and CKF [38]. The SCKF algorithm is outlined below [39];

#### 5.1.1. Time Update

Starting at time instant *k*, assume that the posterior density with the square-root of error covariance matrix is given.

(49) $$p\left(x_{k-1}|Z_{1}^{k-1}\right)=N\left({\hat{x}}_{k-1|k-1}|S_{k-1|k-1}\right)$$

(50) $$P_{k-1|k-1}=S_{k-1|k-1}S_{k-1|k-1}^{⊤}$$

where $P_{k-1|k-1}$ denotes the error covariance matrix, $S_{k-1|k-1}$ is the square-root of the error covariance matrix $P_{k-1|k-1}$, and $Z_{1}^{k-1}$ represents the set of measurements from the start to time instant k−1:

1. Compute the cubature points $X_{i}$:(i=1,2,⋯,m, where *m* = 2$n_{x}$):
  (51) $$X_{i,k-1|k-1}=S_{k-1|k-1}\xi_{i}+{\hat{x}}_{k-1|k-1}$$
  (52) $$\xi_{i}=\sqrt{\frac{m}{2}}{\left[1\right]}_{i}$$
  where $\xi_{i}$ denotes the cubature points weights.
2. Compute the cubature points propagated (i=1,2,⋯,m).
  (53) $$X_{i,k|k-1}^{*}=f\left(X_{i,k-1|k-1}\right)$$
  where f(·) denotes the nonlinear function of the dynamic state equation.
3. Calculate the predicted state.
  (54) $${\hat{x}}_{k|k-1}=\frac{1}{m}\sum_{m=1}^{M}X_{i,k|k-1}^{*}$$
4. Calculate the square-root predicted error covariance.
  (55) $$S_{k|k-1}=\mathrm{Tria}\left(\left[X_{k|k-1}^{*}\;S_{Q,k-1}\right]\right)$$
  (56) $$Q_{k-1}=S_{Q,k-1}S_{Q,k-1}^{⊤}$$
  (57) $$\begin{array}{cc} X_{k|k-1}^{*} & =\frac{1}{\sqrt{m}}[X_{1,k|k-1}^{*}-{\hat{x}}_{k|k-1}\\ & X_{2,k|k-1}^{*}-{\hat{x}}_{k|k-1}\cdots X_{m,k|k-1}^{*}-{\hat{x}}_{k|k-1}] \end{array}$$
  where $S_{Q,k-1}$ is the square-root of process noise covariance $Q_{k-1}$ and $X_{k|k-1}^{*}$ is the weighted-centered matrix. Tria represents the QR-decomposition triangularization algorithm.

#### 5.1.2. Measurement Update

1. Compute the cubature points (i=1,2,⋯,m).
  (58) $$X_{i,k|k-1}=S_{k|k-1}\xi_{i}+{\hat{x}}_{k|k-1}$$
2. Compute the cubature points propagated (i=1,2,...,m).
  (59) $$Z_{i,k|k-1}^{*}=h\left(X_{i,k|k-1}\right)$$
  where h(·) denotes the nonlinear 3D velocity measurement function defined by Equation (39).
3. Calculate the predicted measurement.
  (60) $${\hat{z}}_{k|k-1}=\frac{1}{m}\sum_{m=1}^{M}Z_{i,k|k-1}^{*}$$
4. Compute the square-root innovation covariance matrix.
  (61) $$S_{zz,k|k-1}=\mathrm{Tria}\left(\left[J_{k|k-1}\;S_{R,k}\right]\right)$$
  (62) $$R_{k}=S_{R,k}S_{R,k}^{⊤}$$
  (63) $$\begin{array}{cc} J_{k|k-1} & =\frac{1}{\sqrt{m}}[Z_{1,k|k-1}^{*}-{\hat{z}}_{k|k-1}\\ & Z_{2,k|k-1}^{*}-{\hat{x}}_{k|k-1}\cdots Z_{m,k|k-1}^{*}-{\hat{z}}_{k|k-1}] \end{array}$$
  where $S_{R,k}$ is the square-root of measurement noise covariance $R_{k-1}$ and $J_{k|k-1}$ is the weighted-centered matrix.
5. Compute the cross-covariance matrix.
  (64) $$P_{xz,k|k-1}=X_{k|k-1}J_{k|k-1}^{⊤}$$
  (65) $$\begin{array}{cc} X_{k|k-1} & =\frac{1}{\sqrt{m}}[X_{1,k|k-1}-{\hat{x}}_{k|k-1}\\ & X_{2,k|k-1}-{\hat{x}}_{k|k-1}\cdots X_{m,k|k-1}-{\hat{x}}_{k|k-1}] \end{array}$$
6. Calculate the square-root cubature Kalman gain.
  (66) $$W_{k}=\left(P_{xz,k|k-1}/S_{zz,k|k-1}^{⊤}\right)/S_{zz,k|k-1}$$
7. Compute the updated state estimate.
  (67) $${\hat{x}}_{k|k}={\hat{x}}_{k|k-1}+W_{k}\left(z_{k}-{\hat{z}}_{k|k-1}\right)$$
8. Calculate the square-root estimated error covariance.
  (68) $$S_{k|k}=\mathrm{Tria}\left(\left[X_{k|k-1}-W_{k}J_{k|k-1}\;W_{k}S_{R,k}\right]\right)$$

### 5.2. Interacting Multiple Model Estimator

Since the moving target does not necessarily follow a single dynamic model, modern tracking systems typically use the IMM filter [40] for maneuvering target tracking [29]. If the target dynamics are characterized by multiple switching models (r>1), the posterior density of the target state vector is a mixture density [11,12,25,31]. The objective of the IMM filter is to combine all the mixture components into a single Gaussian distribution in a manner that the first and second moments are matched [24,25,29]. At the current time instant *k*, the IMM algorithm begins with *r* model-conditioned state estimates ${\hat{x}}_{k-1|k-1}^{i}$, square-root error covariance matrix $S_{k-1|k-1}^{i}$, and model probabilities $\mu_{k-1|k-1}^{i}$ at instant k−1[24]. Upon receiving the current measurement $z_{k}$, the IMM algorithm follows the steps outlined below [29,31,41]:

1. Computing mixing probabilities:
  (69) $${\bar{c}}^{j}=\sum_{i=1}^{r}p_{ij}\mu_{k-1|k-1}^{i}$$
  (70) $$\mu_{k-1|k-1}^{\left(i,j\right)}=P\left\{M_{k-1}^{i}|M_{k}^{j},Z_{1}^{k-1}\right\}=\frac{1}{{\bar{c}}^{j}}p_{ij}\mu_{k|k-1}^{i}$$
  where $\mu_{k-1|k-1}^{\left(i,j\right)}$ represents the model probability that model $M^{i}$ was effective at time instant k−1 provided that $M^{j}$ is effective at time instant *k* conditioned on $Z_{1}^{k-1}$. Here, $Z_{1}^{k-1}$ represents the measurement history from start to instant k−1. $p_{ij}$ denotes the elements of model transition probabilities matrix, $\mu_{k-1|k-1}^{i}$ is the model probability for *i*th SCKF, and $\mu_{k-1|k-1}^{\left(i,j\right)}$ is mixture probability for i,j=1,⋯,r.
2. Interaction/mixing of state estimates:
  (71) $${\hat{x}}_{k-1|k-1}^{0j}=\sum_{i=1}^{r}{\hat{x}}_{k-1|k-1}^{i}\mu_{k-1|k-1}^{\left(i,j\right)}$$
  (72) $$P_{k-1|k-1}^{0j}=\sum_{i=1}^{r}\mu_{k-1|k-1}^{\left(i,j\right)}\left\{S_{k-1|k-1}^{i}{\left(S_{k-1|k-1}^{i}\right)}^{⊤}+\left[{\hat{x}}_{k-1|k-1}^{i}-{\hat{x}}_{k-1|k-1}^{0j}\right]{\left[{\hat{x}}_{k-1|k-1}^{i}-{\hat{x}}_{k-1|k-1}^{0j}\right]}^{⊤}\right\}$$
  where ${\hat{x}}_{k-1|k-1}^{0j}$ represents the mixed initial state estimate for the *j*th SCKF, $P_{k-1|k-1}^{0j}$ represents the error covariance corresponding to ${\hat{x}}_{k-1|k-1}^{0j}$, ${\hat{x}}_{k-1|k-1}^{i}$ represents the *i*th SCKF state estimate, and $S_{k-1|k-1}^{i}$ is the square-root covariance matrix for ${\hat{x}}_{k-1|k-1}^{i}$i [41].
3. Updating state estimates: The initial condition state estimate ${\hat{x}}_{k-1|k-1}^{0j}$ and error covariance matrix $P_{k-1|k-1}^{0j}$ for each filter model are used to compute the updated state estimate ${\hat{x}}_{k|k}^{j}$ and square-root error covariance $S_{k|k}^{j}$ for the SCKF model.
4. Computing the model likelihood:
  (73) $$\begin{array}{cc} {\tilde{v}}_{k}^{j} & =z_{k}-h({\hat{x}}_{k|k-1}^{j})\\ \; & =z_{k}-\left[\begin{array}{c} \frac{\left(x_{k}-x_{s}\right)v_{x_{k}}+\left(y_{k}-y_{s}\right)v_{y_{k}}+\left(z_{k}-z_{s}\right)v_{z_{k}}}{\sqrt{{\left(x_{k}-x_{s}\right)}^{2}+{\left(y_{k}-y_{s}\right)}^{2}+{\left(z_{k}-z_{s}\right)}^{2}}}\\ \frac{\left(y_{k}-y_{s}\right)v_{x_{k}}-\left(x_{k}-x_{s}\right)v_{y_{k}}}{{\left(x_{k}-x_{s}\right)}^{2}+{\left(y_{k}-y_{s}\right)}^{2}}\\ \frac{R_{k}^{2}v_{z_{k}}-\left(x_{k}-x_{s}\right)\left(z_{k}-z_{s}\right)v_{x_{k}}-\left(y_{k}-y_{s}\right)\left(z_{k}-z_{s}\right)v_{y_{k}}}{\rho_{k}^{2}R_{k}} \end{array}\right]{\hat{x}}_{k|k-1}^{j} \end{array}$$
  (74) $$\wedge_{k}^{j}=\frac{1}{\sqrt{|2\pi S_{Z,k}^{j}|}}\exp\left\{-0.5{\left[{\tilde{v}}_{k}^{j}\right]}^{T}{\left[S_{Z,k}^{j}\right]}^{-1}\left[{\tilde{v}}_{k}^{j}\right]\right\}$$
  (75) $$S_{Z,k}^{j}=A_{k}^{j}{\left(A_{k}^{j}\right)}^{⊤}$$
  where $\wedge_{k}^{j}$ is the likelihood function and $A_{k}^{j}$ represents the square-root measurement residual covariance matrix for the *j*th SCKF model.
5. Updating the model probability:
  (76) $$c=\sum_{j=1}^{r}\wedge_{k}^{j}{\bar{c}}^{j}$$
  (77) $$\mu_{k|k}^{j}=P\left\{M_{k}^{j}|Z_{1}^{k}\right\}=\frac{1}{c}\wedge_{k}^{j}{\bar{c}}^{j}$$
6. Combining the state mean and covariance estimates for the output:
  (78) $${\hat{x}}_{k|k}=\sum_{j=1}^{r}{\hat{x}}_{k|k}^{j}\mu_{k|k}^{j}$$
  (79) $$\begin{array}{cc} P_{k|k} & =\sum_{j=1}^{r}\mu_{k|k}^{j}\{S_{k|k}^{j}{\left(S_{k|k}^{j}\right)}^{⊤}\\ & +\left[{\hat{x}}_{k|k}-{\hat{x}}_{k|k}^{j}\right]{\left[{\hat{x}}_{k|k}-{\hat{x}}_{k|k}^{j}\right]}^{⊤}\} \end{array}$$

The proposed MTT algorithm utilizes the IMM-SCKF estimator with the 3D velocity measurement function for the confirmed tracks’ state estimation. For the tracks not receiving measurements during the data association process, the predicted state and covariance estimates become the measurement-updated state and covariance estimates. Since only a few tentative tracks fulfill the criteria to be inserted into the confirmed tracks’ list, a simple NCV-SCKF estimator is used for tentative track state estimation. Employing the IMM-SCKF estimator for the targets in the tentative tracks’ list will increase the computational load and complexity for the data processing unit.

## 6. Filter Initialization

Filter initialization is an extremely crucial problem in tracking applications, specifically for nonlinear systems, as nonlinear estimation filters generally depend on approximation theory [24,25]. The nonlinear measurement function in Equation (39), defining the relationship between 3D velocity measurements and the state of the target, makes finding the analytical solution impossible for filter initialization. Therefore, the initial range measurement and initial azimuth and elevation angles determined from Equations (41) and (42), respectively, are used to extract the 3D Cartesian position $\left(x_{0},y_{0},z_{0}\right)$ of the target. That is,

(80) $$x_{0}=\rho \cos\varphi \sin\theta$$

(81) $$y_{0}=\rho \cos\varphi \cos\theta$$

(82) $$z_{0}=\rho \sin\varphi$$

The velocity of the target is calculated by integrating the position estimate at two consecutive time instants, i.e., $v_{x_{0}}=\left(x_{1}-x_{0}\right)⁄\Delta T$, $v_{y_{0}}=\left(y_{1}-y_{0}\right)⁄\Delta T$, and $v_{z_{0}}=\left(z_{1}-z_{0}\right)⁄\Delta T$. Since the azimuth and elevation angles calculated are highly sensitive to the range measurement error, (R,θ,φ) measurements are not reliable for target tracking in the 3D Cartesian space. These measurements are only utilized for filter initialization, and tracking is performed based on the 3D velocity information obtained from the interferometric radar.

## 7. Rule-Based M/N Logic for Track Management

Since the GNN algorithm is incapable of addressing the track management issue automatically, the rule-based M/N logic is used to handle the appearance and disappearance of the targets in the radar’s FOV. The track management involves new track initiation, tentative track confirmation, and tentative/confirmed track deletion. The measurement-to-track data association procedure is followed by the track management step, which is responsible for managing both the tentative tracks’ list and confirmed tracks’ list on the basis of predefined rules. Each track list contains the information of state estimate vector ${\hat{x}}_{k|k}$, square-root error covariance estimate matrix $S_{k|k}$, measurement residual covariance matrix $S_{Z,k}$, “Hit” counter, and “Miss” counter. Upon receiving a new set of 3D velocity measurements from the interferometric radar, these measurements are first tested for association with the confirmed tracks. If a confirmed track does not get associated with a measurement during this step, its “Hit” counter is decremented and its “Miss” counter is incremented. The measurements not associated with the confirmed tracks are tested for data association with existing tentative tracks. The “Hit” counters of the tentative tracks receiving measurements during the data association procedure get incremented, whereas their “Miss” counters remain unchanged. On the other hand, if a tentative track does not receive any measurement during the data association step, its “Hit” counter remains unchanged, whereas its “Miss” counter gets incremented. The still unassociated measurements are utilized by the track management unit to initiate new tentative tracks. The “Hit” and “Miss” counters of new tentative tracks are set to 1 and 0, respectively. The tentative tracks satisfying the 2/2 and 2/3 rules are inserted in the confirmed tracks’ list. This means that a tentative track receiving measurements during the first two consecutive scans of the data and then receiving measurements at least twice during the next three consecutive scans is eligible to be inserted in the confirmed tracks’ list. At this stage, the ”Hit” and “Miss” counters of the confirmed tracks are set as ”Hit = 5” and “Miss = 0”. The tentative track not satisfying the 2/2 and 2/3 criteria is deleted from the tentative track list. If a confirmed track does not receive any measurements during five consecutive scans of data, i.e., “Hit = 0” and “Miss = 5”, the delete flag for this track is set to 1 and the track is deleted from the confirmed tracks’ list [29].

## 8. Performance Evaluation Simulations

This section presents the performance evaluation of the proposed 3D MTT algorithm on the basis of a number of performance evaluation metrics for non-maneuvering and maneuvering multi-target scenarios in the presence of process noise, measurement noise, and clutter due to false alarms.

### 8.1. Performance Metrics

The performance evaluation metrics considered in the proposed research include the following.

#### 8.1.1. Root-Mean-Squared Error in Position

Assume $\left[x_{k},y_{k},z_{k}\right]$ and $\left[{\hat{x}}_{k},{\hat{y}}_{k},{\hat{z}}_{k}\right]$) represent the true and estimated positions, respectively, of a target at time instant *k* in 3D Cartesian coordinates. For *M* number of Monte Carlo runs, the RMSE in the position of the target is defined as [24,29]

(83) $${\mathrm{RMSE}}_{k}^{pos}=\sqrt{\frac{1}{M}\sum_{i=1}^{M}{\left({\hat{x}}_{k}^{i}-x_{k}^{i}\right)}^{2}+{\left({\hat{y}}_{k}^{i}-y_{k}^{i}\right)}^{2}+{\left({\hat{z}}_{k}^{i}-z_{k}^{i}\right)}^{2}}$$

#### 8.1.2. Root-Mean-Squared Error in Velocity

Similarly, if $\left[v_{x_{k}},v_{y_{k}},v_{z_{k}}\right]$ and $\left[{\hat{v}}_{x_{k}},{\hat{v}}_{y_{k}},{\hat{v}}_{z_{k}}\right]$ represent the true and estimated velocities, respectively, of a target, the RMSE in velocity for *M* number of Monte Carlo runs at time instant *k* can be written as

(84) $${\mathrm{RMSE}}_{k}^{vel}=\sqrt{\frac{1}{M}\sum_{i=1}^{M}{\left({\hat{v}}_{x_{k}}^{i}-v_{x_{k}}^{i}\right)}^{2}+{\left({\hat{v}}_{y_{k}}^{i}-v_{y_{k}}^{i}\right)}^{2}+{\left({\hat{v}}_{z_{k}}^{i}-v_{z_{k}}^{i}\right)}^{2}}$$

#### 8.1.3. Mean Execution Time for One Data Scan

The mean execution time $T_{E}$ of the algorithm for one data scan was computed on a laptop computer. The specifications of the computer were 1.9 GHz processor, 4GB RAM, and Windows 10 for Matlab2018. The total execution time was composed of the time for data association $T_{DA}$, filtering for state estimation $T_{F}$, and track management $T_{TM}$ functions [24,29].

(85) $$T_{E}=T_{DA}+T_{F}+T_{TM}$$

#### 8.1.4. IMM Mean Model Probabilities

The IMM mean model probabilities for maneuvering targets reflect how efficiently the IMM algorithm can recognize the different dynamic motion models of the targets and switch between different filter models accordingly [29].

### 8.2. Parameter Selection and Simulated Data Generation

To evaluate the performance of the proposed MTT algorithm, the targets were considered as point sources in the far-field relative to the observing radar. A DF-FMCW interferometric radar with $f_{c_{1}}=6$ GHz and $f_{c_{2}}=24$ GHz was simulated with a bandwidth B=500 MHz and a sweep time T=1 ms. The sampling frequency was set to be $f_{s}=128$ kHz. The receiving antennas Rx1, Rx2, and Rx3, corresponding to $f_{c_{1}}$, were located at (0,0,0), ($-D_{12}$,0,0), and (0,0,$D_{13}$), where $D_{12}=D_{13}=3$ m [29]. The target trajectories were modeled using the NCV and NCT dynamic models with discrete white Gaussian process noise. The straight line motion of the targets followed the NCV model, whereas the maneuvering motion of the targets followed the NCT model. The standard deviations associated with the linear and circular segments of target motion were assumed to be $\sigma_{v_{1}}=0.20$ and $\sigma_{v_{2}}=0.10$. Further, the radial velocity, azimuth angular velocity, and elevation angular velocity measurement error standard deviations were set as $\sigma_{v_{r}}=0.14$ m/s, $\sigma_{\omega_{\theta }}=0.10$ rad/s, and $\sigma_{\omega_{\varphi }}=0.10$ rad/s, respectively. The probability of gate detection was set as $P_{G}=0.99999$. The sampling interval was ΔT=0.02 s [29]. The number of Monte Carlo simulation runs was M=20. The clutter points assumed a Poisson distribution and were uniformly distributed in the measurement region [42]. Each clutter point was composed of (i) a radial velocity measurement distributed in the range of [$v_{r_{min}},v_{r_{max}}$], (ii) an azimuth angular velocity measurement distributed in [$\omega_{\theta_{\min}},\omega_{\theta_{\max}}$], and (iii) an elevation angular velocity measurement distributed in [$\omega_{\varphi_{\min}},\omega_{\varphi_{\max}}$]. The average number of clutter points was set as 5 for each scan [29]. For the IMM filter with Model 1 as the NCV model and Model 2 as the NCT model, the model transition probabilities matrix is

(86) $$\begin{array}{c} \pi =\left[\begin{array}{cc} 0.99 & 0.01\\ 0.01 & 0.99 \end{array}\right] \end{array}$$

The initial model probability for each filter model was chosen to be $\mu_{j}=0.5$, where j=1,2 [29].

### 8.3. Scenario 1: Two Non-Maneuvering Closely Spaced Targets

**Figure 3:**
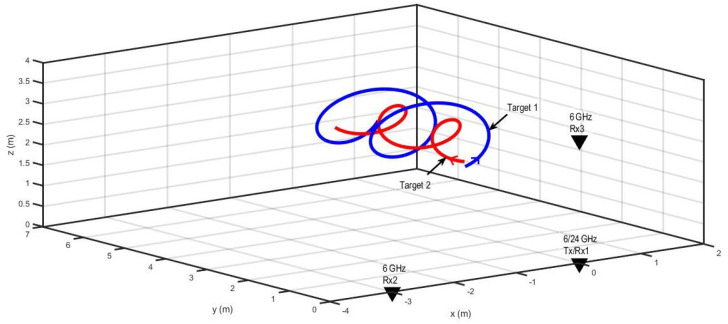
The radar and target geometry for Scenario 1.

Scenario 1 for 3D tracking with two non-maneuvering targets and radar antennas is shown in Figure 3. Table 1 presents the initial states of the targets. The trajectories of the targets were modeled using the NCT dynamic model defined by Equation (23). However, the circular motion was along the xz-plane, rather than the xy-plane, as described by state transition matrix $F_{k-1}^{\mathrm{NCT},3D}$ in Equation (25).

**Figure 4:**
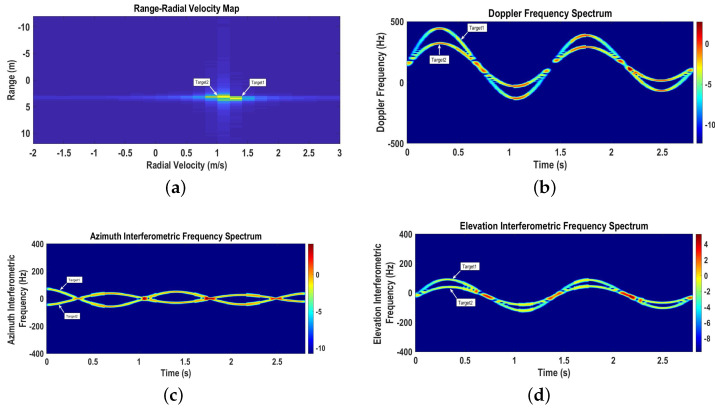
Range–radial velocity map, time-varying Doppler, and azimuth interferometric and elevation interferometric spectrograms for Scenario 1. (**a**) Range–radial velocity map; (**b**) Doppler spectrogram; (**c**) Azimuth interferometric spectrogram; (**d**) Elevation interferometric spectrogram.

The range–radial velocity map ($f_{c_{2}}=24$ GHz) represented by Figure 4a shows two targets with initial ranges of $R_{01}=3.47$ m and $R_{02}=3.11$ m and initial radial velocities of $v_{r_{1}}=1.31$ m/s and $v_{r_{2}}=1.1$ m/s, respectively.

The time-varying Doppler spectrogram ($f_{c_{2}}=24$ GHz), azimuth interferometric spectrogram ($f_{c_{1}}=6$ GHz), and elevation interferometric spectrogram ($f_{c_{1}}=6$ GHz) are shown in Figure 4b–d, respectively. The instantaneous Doppler frequency shift caused by the radial velocity of the target increases for the target moving away from the radar and decreases for the target moving towards the radar. Moreover, the sinusoidal patterns of the radial velocities represent the circular motions of the targets moving towards and away from the radar. The instantaneous azimuth angular frequency caused by the azimuth angular velocity is positive for the target moving along the positive *x* direction and negative for the target moving along the negative *x* direction. Similarly, the instantaneous elevation angular frequency caused by the elevation angular velocity is positive for the target moving in the positive φ direction and negative for the target moving in the negative φ direction.

**Figure 5:**
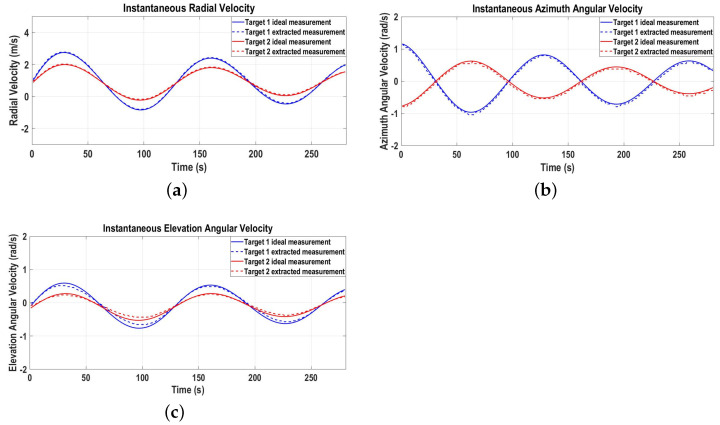
Three-dimensional velocity measurements for Scenario 1. (**a**) Radial velocity measurement; (**b**) Azimuth angular velocity measurement; (**c**) Elevation angular velocity measurement.

Figure 5a–c represent the ideal and extracted radial, azimuth angular, and elevation angular velocities of the targets, providing complete 3D velocity measurement information. The extracted 3D velocity measurements are fed to the proposed GNN-IMM-SCKF algorithm for 3D tracking, which includes data association, state estimation, and track management functions.

**Figure 6:**
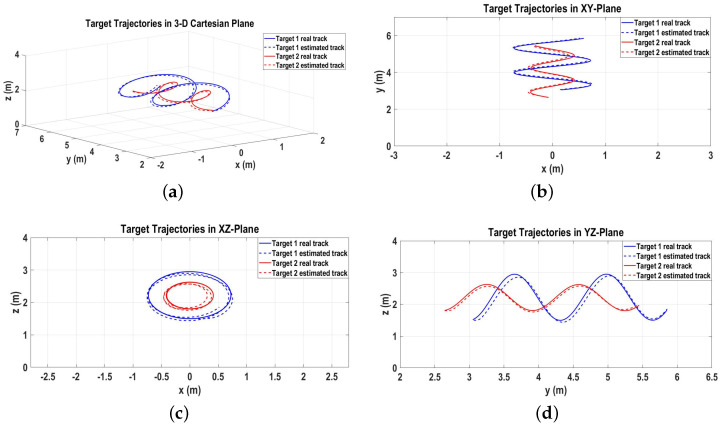
Real and estimated target tracks for Scenario 1. (**a**) Real and estimated target tracks in 3D Cartesian space; (**b**) Real and estimated target tracks in xy-plane; (**c**) Real and estimated target tracks in xz-plane; (**d**) Real and estimated target tracks in yz-plane.

The real target tracks following Equation (23) and the output of the proposed algorithm as estimated target tracks in the 3D Cartesian space are shown in Figure 6a. Moreover, Figure 6b–d represent the target tracks in the 2D xy, xz, and yz Cartesian spaces, respectively.

**Figure 7:**
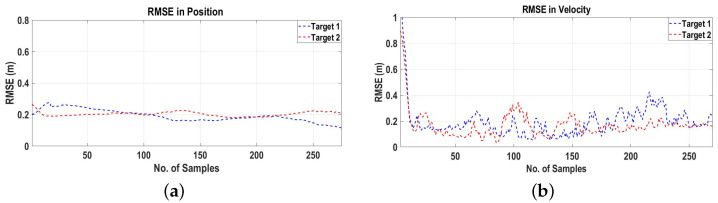
RMSEs in position and velocity for Scenario 1. (**a**) RMSE in position; (**b**) RMSE in velocity.

To access the performance of the proposed algorithm, the RMSEs in the positions and velocities of the targets are plotted in Figure 7a,b, respectively, which prove that the proposed algorithm based on 3D velocity measurements from the interferometric radar can be used for MTT in the 3D Cartesian space.

### 8.4. Scenario 2: Two Maneuvering Closely Spaced Targets

**Figure 8:**
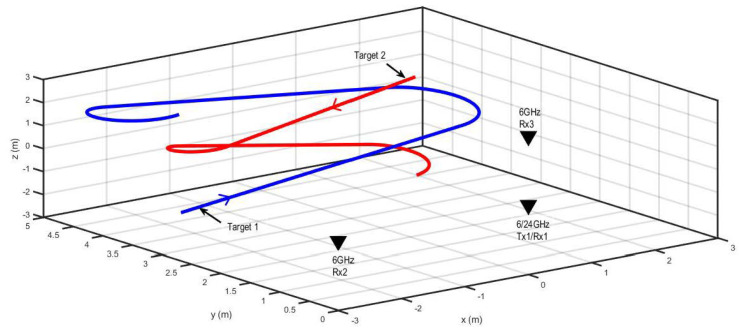
The radar and target geometry for Scenario 2.

Scenario 2 for 3D target tracking with two closely spaced maneuvering targets and radar location is shown in Figure 8. The trajectories of the targets were modeled by the NCV (Equation (18)) and NCT (Equation (23)) dynamic models. The initial states of the targets are summarized in Table 2.

**Figure 9:**
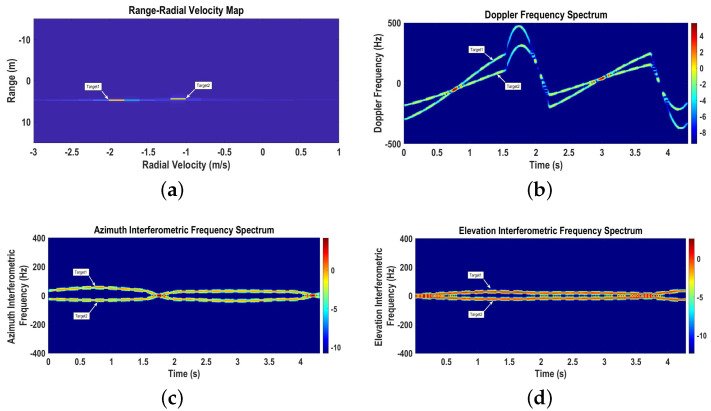
Range–radial velocity map, time-varying Doppler, and azimuth interferometric and elevation interferometric spectrograms for Scenario 2. (**a**) Range–radial velocity map; (**b**) Doppler spectrogram; (**c**) Azimuth interferometric spectrogram; (**d**) Elevation interferometric spectrogram.

The range–radial velocity map plotted in Figure 9a clearly shows two different targets in the radar’s FOV with initial ranges of $R_{01}=4.68$ m and $R_{02}=4.35$ m and initial radial velocities of $v_{r_{1}}=-1.91$ m/s and $v_{r_{2}}=-1.11$ m/s, respectively.

**Figure 10:**
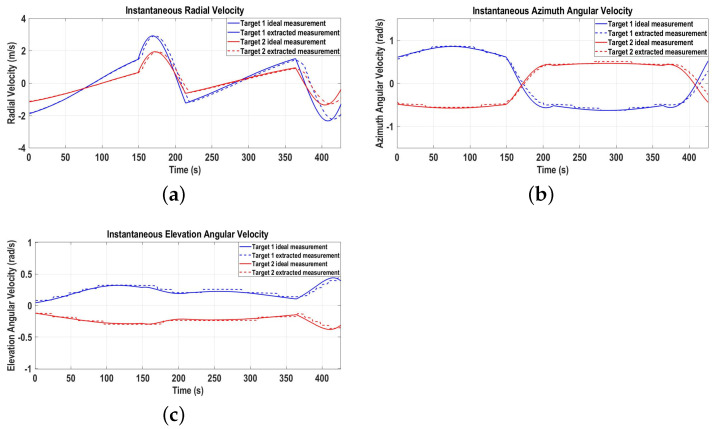
Three-dimensional velocity measurements for Scenario 2. (**a**) Radial velocity measurement; (**b**) Azimuth angular velocity measurement; (**c**) Elevation angular velocity measurement.

Time-varying Doppler, azimuth interferometric, and elevation interferometric spectrograms for Scenario 2 are shown in Figure 9b–d, respectively. The radial, azimuth angular, and elevation angular velocities extracted from the time-varying spectrograms are presented in Figure 10a–c, respectively, together with the ideal 3D velocity measurements. For targets moving towards the radar, the radial velocities decrease, and for targets moving away from the radar, the radial velocities increase. Similarly, the azimuth and elevation angular velocities of the targets moving in positive θ and φ are positive, respectively, and vice versa.

**Figure 11:**
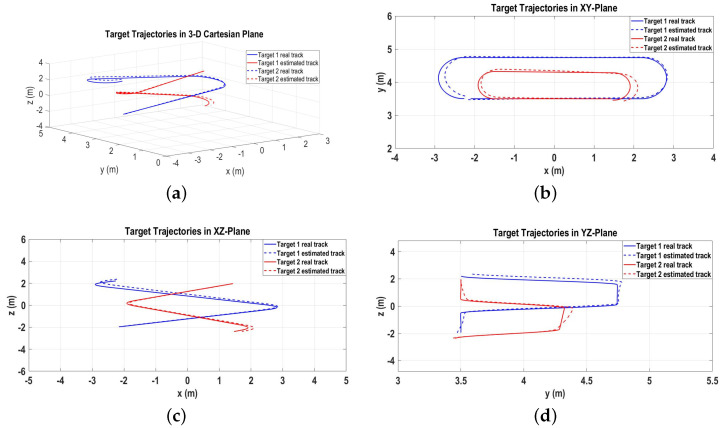
Real and estimated target tracks for Scenario 2. (**a**) Real and estimated target tracks in 3D Cartesian space; (**b**) Real and estimated target tracks in xy-plane; (**c**) Real and estimated target tracks in xz-plane; (**d**) Real and estimated target tracks in yz-plane.

The real target tracks and the estimated target tracks obtained as the output of the GNN-IMM-SCKF algorithm in the 3D Cartesian space are plotted in Figure 11a. The real and estimated 2D tracks in the xy, xz, and yz Cartesian spaces are shown in Figure 11b–d, respectively.

**Figure 12:**
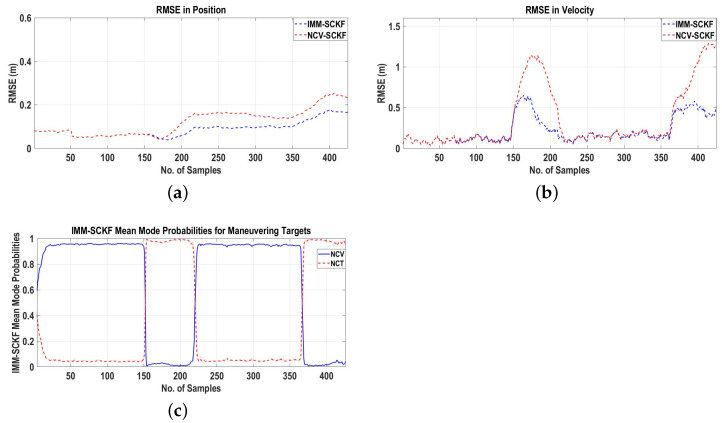
RMSEs in position and velocity and IMM-SCKF mean model probabilities for maneuvering targets for Scenario 2. (**a**) RMSE in position; (**b**) RMSE in velocity; (**c**) IMM-SCKF mean model probabilities.

For the performance evaluation, the RMSEs in the positions and velocities of the targets are presented in Figure 12a,b. Figure 12a,b assert the fact that the RMSEs for the GNN-IMM-SCKF algorithm are less compared to the RMSEs for the GNN-NCV-SCKF algorithm during the targets’ maneuvers. The IMM mean model probabilities for the NCV and NCT dynamic models presented in Figure 12c validate the fact that the proposed GNN-IMM-SCKF algorithm based on 3D velocity measurements obtained from the interferometric radar is applicable to 3D tracking systems for state estimation of maneuvering targets.

The execution times for different 3D target tracking scenarios are summarized in Table 3. It is evident that the data association block of the tracking algorithm consumes more time than the filtering and track management blocks, which is approximately 48–56% of the total execution time. Furthermore, the execution time for the GNN-IMM-SCKF algorithm is approximately 27% greater than the execution time for the GNN-NCV-SCKF algorithm. The total execution time $T_{E}$ being less than the measurement sampling interval ΔT proves the fact that the proposed MTT algorithm can be implemented for real-time applications.

## 9. Conclusions

MTT typically requires either a network of Doppler radar receivers at different locations or a single phased array radar. However, Doppler radar networks have high computational complexity and data throughput attributed to multiple receivers in the network. Moreover, array signal processing techniques for phased array radar are computationally expensive. To resolve the issue, this paper presented an algorithm for detection and tracking of multiple moving targets based on 3D velocity measurements obtained from a dual-orthogonal baseline interferometric radar. First, we presented the mathematical model of the 3D velocities of multiple moving point sources as targets. The radial, azimuth angular, and elevation angular velocities were extracted using reference receiving antenna, a baseline along the x-axis and a second orthogonal baseline along the z-axis, respectively. Then, we derived the nonlinear 3D velocity measurement function, which defines the relationship between the 3D velocity measurements and the state of the target. Based on the 3D velocity measurement function, we introduced the design and implementation of an MTT algorithm, which included the GNN for data association, the IMM-SCKF estimator for state estimation, and the rule-based M/N logic for track management. Then, we performed Monte Carlo simulations for different multi-target scenarios and evaluated the performance of the algorithm in terms of the RMSEs in position and velocity, the mean execution time, and the IMM mean model probability. In order to simulate a multi-target scenario close to a real environment, process noise in the dynamic motion models of the targets was modeled as the DWNA, taking into consideration small accelerations as noise. Moreover, zero-mean Gaussian measurement noise and clutter due to false alarms following a Poisson distribution were added to the received data. Consequently, the simulation results proved the fact that the proposed algorithm is robust and can be applied to practical 3D tracking systems.

## Figures and Tables

**Table 1:** Scenario 1.

Targets				Initial States of Targets			
	x(m)	$v_{x}1(m/s)$	y(m)	$v_{y}(m/s)$	z(m)	$v_{z}(m/s)$	Ω(rad/s)
1	0	3.5	3	1	1.5	0	4.83
2	0	−2	2.6	1	1.8	0	−4.83

**Table 2:** Scenario 2.

Targets				Initial States of Targets			
	x(m)	$v_{x}1(m/s)$	y(m)	$v_{y}(m/s)$	z(m)	$v_{z}(m/s)$	Ω(rad/s)
1	−2.25	3	3.5	0	−2	1	4.83
2	1.5	−2	3.5	0	2	−1	−4.83

**Table 3:** Execution times.

Scenario	Execution Time			
	$T_{E}(\mathrm{ms})$	$T_{\mathrm{DA}}(\mathrm{ms})$	$T_{F}(\mathrm{ms})$	$T_{\mathrm{TM}}(\mathrm{ms})$
1 (GNN-IMM-SCKF)	17.7	10 (56%)	5.94 (34%)	1.80 (10%)
2 (GNN-NCV-SCKF)	15.1	7.25 (48%)	4.41 (29%)	3.44 (23%)
2 (GNN-IMM-SCKF)	19.2	9.18 (48%)	7.53 (39%)	2.48 (13%)

## Data Availability

All data available upon request.
